# Performance evaluation of sodium-ion batteries for microgrid and off-grid energy storage applications

**DOI:** 10.3389/fchem.2026.2007977

**Published:** 2026-10-27

**Authors:** Xingyu Hu

**Affiliations:** Material Science and Engineering, Imperial College London, London, United Kingdom

**Keywords:** battery degradation, microgrid, off-grid systems, sodium-ion batteries, stationary energy storage

## Abstract

**Introduction:**

Sodium-ion batteries are increasingly considered for stationary energy storage; however, their application-level performance depends on how experimentally measured electrochemical behavior translates into realistic grid-connected and standalone operating conditions.

**Methods:**

This study evaluated 24 5.0-Ah sodium-ion pouch cells through baseline electrochemical characterization, identification of a second-order Thevenin electrochemical-thermal model, annual system simulation, and application-specific aging tests. Discharge behavior was characterized at rates of 0.20–2.00 C and temperatures of 10–40 °C. Pulse and impedance measurements were used to parameterize the model across 10%–90% state of charge. The calibrated model was scaled to a 504-kWh grid-connected microgrid and a 120.96-kWh standalone off-grid system, both simulated over 365 days at 15-min resolution.

**Results:**

Increasing discharge rate reduced usable capacity and energy efficiency, whereas Coulombic efficiency remained above approximately 99%. The model reproduced measured voltage responses with millivolt-scale prediction errors and captured state-of-charge- and temperature-dependent resistance trends. The grid-connected microgrid achieved 91.6% round-trip efficiency, an 18.7-kW tracking root-mean-square error, and 4.3% renewable curtailment. The standalone system achieved 89.8% round-trip efficiency, a 1.6% annual loss-of-power-supply probability, and 6.4% curtailment. After 500 equivalent full cycles, capacity state of health was 85.2%, 81.9%, and 79.8% under reference, microgrid, and off-grid cycling, respectively, with corresponding resistance growth of 39%, 58%, and 74%.

**Discussion:**

These findings demonstrate that application-specific duty cycles materially influence the efficiency, reliability, and degradation of sodium-ion battery storage systems. Integrating experimentally calibrated electrochemical-thermal models with application-level simulations provides a practical basis for evaluating battery performance under realistic stationary energy-storage conditions.

## 1 Introduction

The expansion of variable renewable generation has increased the need for electrochemical storage systems capable of providing short-term power balancing, peak reduction, energy shifting, and reliability support across both grid-connected and autonomous power networks. In microgrids, the value of battery storage is determined not only by installed energy capacity but also by the interaction among state-of-charge constraints, converter operation, dispatch logic, and the temporal mismatch between generation and demand (Chaudhary et al., 2021; Chen et al., 2019). For remote and off-grid systems, this interaction is more restrictive because insufficient storage or inadequate backup coordination directly affects supply reliability and renewable-energy utilization (Javed et al., 2022). Consequently, evaluating an emerging battery chemistry for stationary use requires linking cell-level electrochemical behavior to the dynamic duty cycles imposed by the intended energy-system application rather than relying exclusively on nominal material properties or laboratory cycling under constant current.

Sodium-ion batteries (SIBs) have emerged as a technically credible option for stationary storage because sodium resources are widely distributed and several cell components can be based on comparatively abundant elements. Early full-cell analyses emphasized that the practical competitiveness of SIBs depends on balanced electrode capacities, first-cycle losses, voltage efficiency, and full-cell rather than half-cell behavior (Chen et al., 2019). Subsequent assessments have reinforced the relevance of SIBs for large-scale storage while showing that commercialization depends on simultaneous progress in energy density, cycle life, rate capability, interfacial stability, manufacturing scalability, and cost (Narayan Singh et al., 2023). A long-term perspective on the technology further indicates that the transition from electrode-level demonstrations to robust full cells is now central to evaluating whether laboratory advances can translate into useful energy-storage devices (Gao et al., 2024). A recent comprehensive assessment likewise identifies electrode stability, electrolyte compatibility, manufacturability, and application-specific performance as persistent constraints on wider deployment (Phogat et al., 2025).

Among positive-electrode families, layered sodium transition-metal oxides remain attractive because of their relatively high operating voltage, substantial reversible capacity, and compatibility with scalable synthesis routes. However, phase transitions, air sensitivity, structural rearrangement at high states of charge, and composition-dependent voltage decay can compromise long-term stability. Process-control studies have therefore highlighted the importance of synthesis conditions and particle-level engineering for achieving practically relevant layered-oxide cathodes (Lamb and Manthiram, 2020). More recent techno-electrochemical analyses have shown that layered oxides can be competitive when storage mechanism, material cost, voltage window, and full-cell design are considered together (Zuo et al., 2023). Importantly, ampere-hour-scale pouch-cell demonstrations now show that appropriately controlled layered-oxide structures can sustain reversible electrochemical behavior at cell formats more representative of practical deployment (Wang et al., 2024). Recent studies further show that layered-oxide cycling stability is strongly coupled to phase-transition control, elemental substitution, and synthesis route, reinforcing the need to interpret resistance growth together with possible cathode structural evolution (An et al., 2024; Zeng et al., 2025; Li et al., 2025).

Hard carbon is the leading anode candidate for commercial SIBs because its disordered structure provides sodium-storage sites over both sloping and low-voltage plateau regions while avoiding the poor sodium intercalation behavior of conventional graphite. Nevertheless, sodium-storage mechanisms, pore architecture, surface area, irreversible sodium consumption, and initial Coulombic efficiency remain strongly coupled to practical full-cell performance (Zhao et al., 2021). Recent reviews have emphasized that morphology, defect density, heteroatom chemistry, and graphitic-domain organization must be controlled to balance capacity, rate capability, and cycling stability (Shao et al., 2022). More recent synthesis-to-performance analyses further reinforce the need to coordinate pore structure, precursor chemistry, and carbon microstructure when targeting commercially relevant hard-carbon behavior (Wu C. et al., 2024). The electrolyte–hard-carbon interface is equally important because solvent chemistry and additive selection govern solid-electrolyte-interphase formation, impedance growth, and irreversible charge consumption (Liu et al., 2022). Interphase-focused studies further indicate that SEI composition and evolution remain major determinants of efficiency retention and long-term anode stability (Meng et al., 2022). At the device level, advances in hard-carbon design have enabled fast-charging sodium-ion cells under increasingly practical conditions, demonstrating that rate performance can be improved without necessarily sacrificing long-term cycling stability (Li et al., 2024). Recent hard-carbon studies likewise emphasize that initial Coulombic efficiency, pore architecture, and interfacial/electronic-state control govern irreversible sodium loss and impedance development (Zheng et al., 2024; Xie et al., 2026).

Electrolyte selection is therefore a central component of practical SIB design. Carbonate-based electrolytes remain attractive because they are compatible with established manufacturing infrastructure, but their performance depends on controlling solvent decomposition and stabilizing both anode and cathode interfaces. Low-solvation electrolyte concepts have demonstrated that suppressing dissolution of interphase species can markedly improve the stability of high-voltage sodium-ion full cells (Jin et al., 2022). Related work on initial Coulombic efficiency has shown that interfacial engineering, electrolyte optimization, and sodium-compensation strategies can reduce sodium inventory loss and improve usable full-cell energy (Wan et al., 2023). Full-cell presodiation has likewise been demonstrated as a means of compensating sodium deficiency while promoting more stable electrode interfaces (Wang et al., 2023). These findings are directly relevant to stationary storage because efficiency losses and progressive impedance rise translate into lower usable energy, greater thermal generation, and reduced lifetime at the system level.

The environmental and economic case for SIBs is similarly application dependent. Life-cycle modeling has shown that sodium-ion chemistries can reduce reliance on critical raw materials, although their environmental advantage is sensitive to cell composition, energy density, manufacturing energy, cycle life, and recycling assumptions (Peters et al., 2021). Manufacturing-stage assessments also indicate that electricity mix and process energy can materially influence comparative carbon emissions, so resource abundance alone does not guarantee a lower environmental burden (Lai et al., 2023). Prospective studies based on abundant-element chemistries nevertheless show a favorable pathway for reducing mineral-resource scarcity impacts as SIB production scales (Wickerts et al., 2024). Additional life-cycle comparisons confirm that performance improvements at the cell level can be as important as background-grid decarbonization in determining future climate impacts (Batuecas et al., 2024). Recent techno-economic roadmap analysis further suggests that sodium-ion competitiveness with lithium-ion technology will depend on coordinated improvement in material cost, energy density, cycle life, manufacturing scale, and supply-chain conditions rather than on a single intrinsic advantage (Yao et al., 2025).

For microgrid operation, batteries are commonly dispatched to attenuate rapid net-load fluctuations, reduce peak exchange with the utility grid, and absorb excess renewable generation. Reviews of battery scheduling in microgrids show that SOC limits, power constraints, degradation, and converter losses must be represented explicitly when evaluating dispatch performance and decarbonization benefits (Lipu et al., 2022). At the grid-integration level, electrochemical storage can mitigate renewable-power variability, but the suitability of a battery technology depends on its response time, efficiency, durability, and energy-to-power characteristics under the targeted service (Deguenon et al., 2023). In isolated systems, the operating problem is different: renewable generation must be used preferentially while the battery and dispatchable backup generation jointly maintain adequacy. Stand-alone photovoltaic–wind–battery–diesel studies demonstrate that storage and backup sizing strongly affect fuel use, renewable penetration, and reliability (Boujdaini et al., 2022). Remote-microgrid analyses similarly identify curtailment, load loss, and storage utilization as coupled performance indicators rather than independent outcomes (Javed et al., 2022).

Despite rapid progress in SIB materials and cell design, a methodological gap remains between electrochemical characterization and application-level performance assessment. Much of the SIB literature still evaluates electrodes or cells under standardized galvanostatic protocols, whereas power-system studies frequently assume generic battery parameters that are not directly derived from the chemistry being simulated. Recent overviews have therefore emphasized the need for full-cell evaluation and stronger links between electrochemical mechanisms and energy applications (Wu Y. et al., 2024). Plant-derived and other hard-carbon studies also illustrate that performance measured in simplified half-cell configurations cannot be transferred directly to practical full cells without accounting for sodium inventory and electrode balancing (Alvira et al., 2022). This gap becomes particularly important when comparing grid-connected and off-grid duty cycles, because shallow high-frequency cycling, extended discharge intervals, temperature effects, converter losses, and backup-generation thresholds impose substantially different electrochemical stresses. A chemistry-specific framework that preserves experimentally measured voltage, resistance, thermal, efficiency, and aging behavior across system simulations is therefore needed for a defensible application-level comparison.

The present study addresses this need through a controlled laboratory-plus-system evaluation of 5.0-Ah sodium-ion pouch cells using a layered-oxide cathode and hard-carbon anode. Baseline characterization spans 0.20–2.00 C discharge rates and 10 °C–40 °C operation, while pulse and impedance measurements are used to identify and validate a second-order Thevenin electrochemical-thermal model. The experimentally calibrated model is then scaled without substituting lithium-ion surrogate parameters to a 504-kWh grid-connected microgrid and a 120.96-kWh standalone off-grid system, both simulated over a common 365-day horizon at 15-min resolution. Application-specific aging is evaluated through reference, microgrid, and off-grid duty-cycle groups with reference performance tests every 100 equivalent full cycles up to 500 EFC or 80% capacity SOH. This design enables electrochemical efficiency, voltage-model error, resistance growth, capacity fade, round-trip efficiency, renewable curtailment, microgrid tracking error, and off-grid loss-of-power-supply probability to be interpreted within one internally consistent parameter framework.

### 1.1 Contribution of the present study

The contribution of this work is an integrated, chemistry-specific evaluation framework that connects measured sodium-ion pouch-cell behavior to two distinct stationary-storage applications. Specifically, the study (i) derives the electrical, thermal, efficiency, and aging parameters used in the system simulations from the same experimentally characterized sodium-ion cell set; (ii) validates a second-order Thevenin model across SOC and temperature before applying it to annual microgrid and off-grid simulations; (iii) compares grid-connected net-load smoothing with renewable-first standalone operation under application-specific converter, SOC, and backup-generation constraints; and (iv) evaluates long-term degradation using reference, microgrid, and off-grid duty cycles with repeated capacity, pulse, and impedance measurements to 500 EFC. This structure permits system-level performance and durability to be compared without introducing lithium-ion surrogate parameters or disconnecting application metrics from the underlying sodium-ion electrochemistry.

## 2 Methods

The study was designed as a controlled laboratory-plus-system evaluation integrating electrochemical characterization, dynamic model identification, application-specific duty-cycle emulation, and annual energy-system simulation within a single internally consistent parameter set. The primary objective was to determine whether the selected sodium-ion cell chemistry could maintain usable energy, power capability, efficiency, and electrochemical stability under duty patterns representative of grid-connected microgrids and standalone off-grid systems. Cell-level measurements supplied the voltage, resistance, thermal, state-of-charge (SOC), and aging parameters used in the application simulations; no lithium-ion surrogate electrochemical parameters were introduced. The integrated procedure is summarized in Figure 1.

**FIGURE 1:**
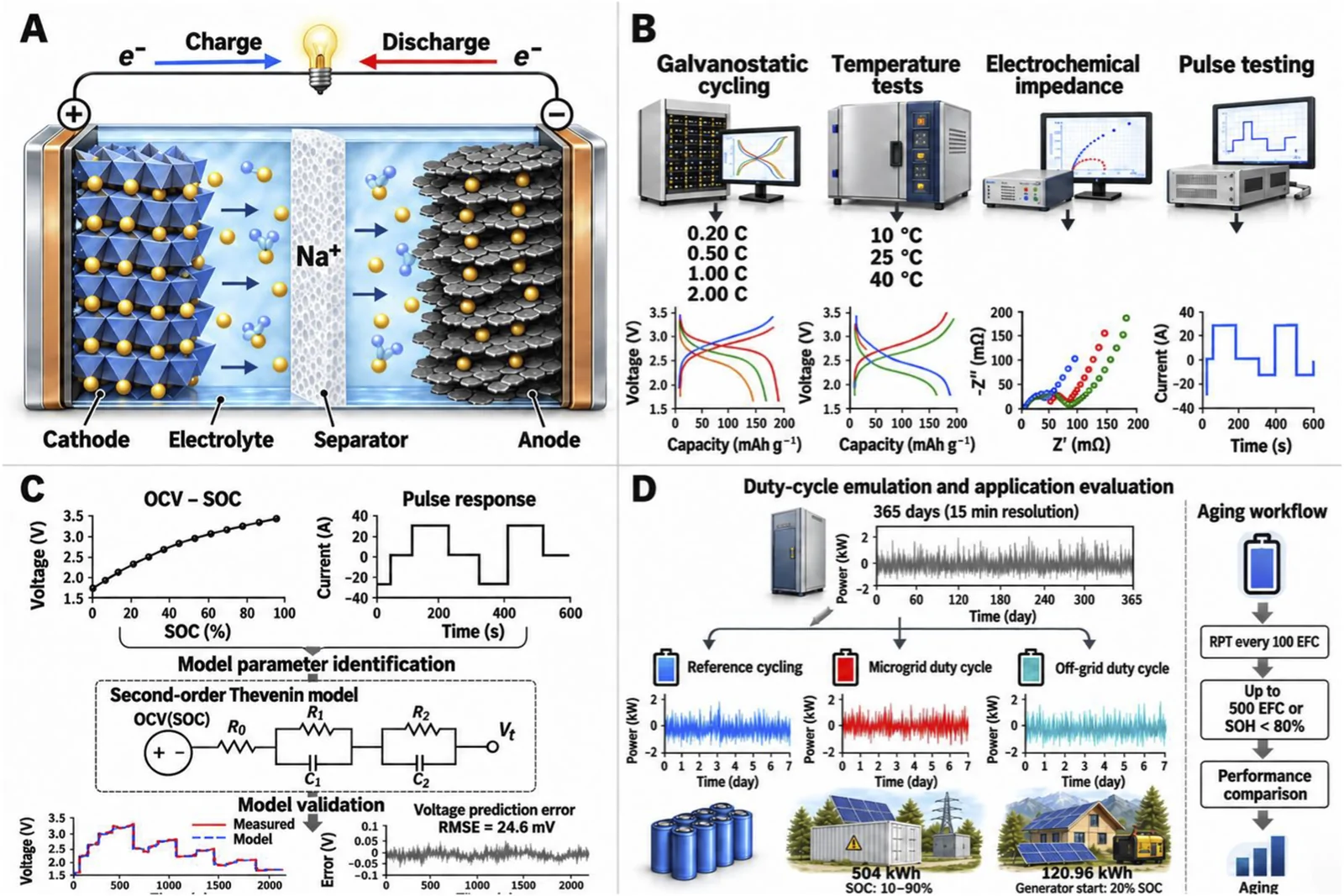
Integrated workflow for sodium-ion battery evaluation: **(A)** Battery operating principle and components; **(B)** Experimental electrochemical characterization; **(C)** Equivalent-circuit model identification and validation; and **(D)** Duty-cycle simulation and application-specific aging assessment.

### 2.1 Cell chemistry, fabrication, and baseline electrochemical characterization

Twenty-four 5.0-Ah laminate pouch cells were considered. The positive electrode used a layered sodium transition-metal oxide, ${\text{Na}}_{0.67}{\text{Fe}}_{0.50}{\text{Mn}}_{0.50}O_{2}$ , with a dry composition of 92 wt% active material, 4 wt% conductive carbon, and 4 wt% poly (vinylidene fluoride). The negative electrode contained 93 wt% hard carbon, 2 wt% conductive carbon, 2 wt% carboxymethyl cellulose, and 3 wt% styrene-butadiene rubber. The nominal cathode areal loading was 17.5 mg cm^-2^, the designed negative-to-positive capacity ratio was N/P=1.10 , and the nominal cell voltage and capacity were 3.0 V and 5.0 A h, respectively. The electrolyte was 1.0 mol L^-1^ ${\text{NaPF}}_{6}$ in propylene carbonate/ethyl methyl carbonate (30:70 by volume) containing 2 wt% fluoroethylene carbonate; electrolyte filling was fixed at 3.0 g Ah^-1^. A 20- μ m polyolefin separator and aluminum current collectors were used on the positive side, whereas aluminum foil was used for the hard-carbon negative electrode to exploit the absence of Na-Al alloying within the selected potential window.

The N/P capacity ratio of 1.10 was selected as a modest hard-carbon excess that preserved cathode utilization while reducing the risk of local anode sodium saturation near the upper charge limit. A balancing sensitivity calculation at N/P = 1.05, 1.10, and 1.15, with all other parameters fixed, was also performed to quantify the effect of electrode balance on 500-EFC capacity and resistance outcomes.

After electrolyte filling, cells were rested for 12 h at 25 °C ± 1 °C and subjected to three formation cycles at 0.10 C between 2.00 and 3.65 V. A constant-current/constant-voltage charge protocol was used, with the constant-voltage step terminated at 0.05 C as described by Equation 1. Cells were then rested for 24 h before baseline testing. Twelve cells were reserved for electrochemical characterization and model identification; the remaining twelve were randomized into three four-cell aging groups: a constant-current reference group, a microgrid duty-cycle group, and an off-grid duty-cycle group. The principal cell and test settings are listed in Table 1.

$$Q_{\text{dis}}=\frac{1}{3600}\int_{t_{0}}^{t_{f}}I\left(t\right)\;dt$$ (1)

where $Q_{\text{dis}}$ is the discharge capacity in Ah, $I\left(t\right)$ is cell current in A, and t is time in s. The corresponding discharge energy was obtained from the simultaneously sampled terminal voltage $V\left(t\right)$ Equation 2:

$$E_{\text{dis}}=\frac{1}{3600}\int_{t_{0}}^{t_{f}}V\left(t\right)I\left(t\right)\;dt$$ (2)

**TABLE 1:** Sodium-ion cell configuration and laboratory electrochemical test conditions.

Item	Specification
Cell format and nominal rating	Laminate pouch; 5.0 A h; 3.0 V nominal
Cathode	${\text{Na}}_{0.67}{\text{Fe}}_{0.50}{\text{Mn}}_{0.50}O_{2}$ ; 92:4:4 active/carbon/pvdf
Anode	Hard carbon; 93:2:2:3 active/carbon/cmc/sbr
Voltage window	2.00–3.65 V
Electrolyte	1.0 mol L^-1^ ${\text{NaPF}}_{6}$ in PC/EMC (30:70 vol%) + 2 wt% FEC
Formation	3 cycles at 0.10 C, 25 °C ± 1 °C
Characterization temperatures	10, 25, and 40 °C; 3 h equilibration before testing
Rate-capability sequence	0.20, 0.50, 1.00, and 2.00 C discharge; 0.50 C charge
Pulse test	±1.0 C, 10 s pulse +50 s relaxation, SOC = 10–90% in 10% steps
EIS	100 kHz–10 mHz; 10 mV perturbation; 50% SOC
Aging allocation	References, microgrid, off-grid; n=4 cells per group

Discharge capacity was calculated by integrating the measured positive discharge current over the interval bounded by the voltage limits.

Coulombic efficiency and energy efficiency were then computed as Equations 3, 4:

$$\eta_{C}=\frac{Q_{\text{dis}}}{Q_{\text{ch}}}\times 100$$ (3)

$$\eta_{E}=\frac{E_{\text{dis}}}{E_{\text{ch}}}\times 100$$ (4)

where $Q_{\text{ch}}$ and $E_{\text{ch}}$ denote charge capacity and charge energy, respectively. Rate capability was evaluated after a 0.50 C reference cycle at 25 °C. Each discharge rate was repeated twice, and the second cycle was used for comparison to minimize transient conditioning effects. Open-circuit-voltage (OCV) data were obtained by charging or discharging to 10% SOC increments, followed by a 60-min rest. Electrochemical impedance spectroscopy (EIS) was conducted at 50% SOC after thermal equilibration at 10, 25, and 40 °C. The same EIS condition was repeated during reference performance tests (RPTs) to track changes in ohmic and polarization resistance.

### 2.2 Dynamic electrochemical-thermal model and parameter identification

A second-order Thevenin equivalent-circuit model was used because it can reproduce the fast ohmic response and the two dominant polarization time scales while remaining computationally efficient for annual microgrid simulations. SOC is denoted by $z\in \left[0,1\right]$ . The equilibrium OCV at 25 °C was represented by a cubic function fitted to the measured rest data:

A cubic OCV relation was retained because it was the lowest-order polynomial that reproduced the measured monotonic curvature across 10%–90% SOC without additional turning points. Fit quality was assessed using overall RMSE and the absolute residuals at the 10% and 90% SOC nodes (Equation 5).

$$U_{\text{oc}}\left(z\right)=2.10+1.55z-0.85z^{2}+0.80z^{3}$$ (5)

The polynomial was used only inside the experimentally validated SOC interval of 0.10–0.90. Temperature dependence was included through the measured reversible voltage coefficient $\kappa_{U}=-4.5\times 10^{-4}$ V K^−1^ Equation 6:

$$U_{\text{oc}}\left(z,T\right)=U_{\text{oc}}\left(z\right)+\kappa_{U}\left(T-298.15\right)$$ (6)

With discharge current defined as positive, terminal voltage was calculated as Equation 7:

$$V_{t}=U_{\text{oc}}\left(z,T\right)-IR_{0}-V_{1}-V_{2}$$ (7)

where $R_{0}$ is the instantaneous resistance and $V_{1}$ and $V_{2}$ are the polarization voltages associated with the $\left(R_{1},C_{1}\right)$ and $\left(R_{2},C_{2}\right)$ branches. The discrete polarization-state update for $j\in \left\{1,2\right\}$ was Equation 8:

$$V_{j,k+1}=e^{-\Delta t/\left(R_{j}C_{j}\right)}V_{j,k}+R_{j}\left(1-e^{-\Delta t/\left(R_{j}C_{j}\right)}\right)I_{k}$$ (8)

and the cell SOC update was Equation 9:

$$z_{k+1}=z_{k}-\frac{\xi \left(I_{k}\right)I_{k}\Delta t}{3600Q_{nom}}$$ (9)

where $Q_{nom}=5.0$ Ah and $\xi \left(I_{k}\right)=1$ during discharge and $\xi \left(I_{k}\right)=\eta_{\text{ch}}$ during charge, with $\eta_{\text{ch}}=0.995$ . Resistance-capacitance values were identified independently at each SOC and temperature node from the pulse-relaxation responses and then bilinearly interpolated within the experimentally sampled 10%–90% SOC and 10°C–40 °C domain during simulation; extrapolation beyond this domain was not permitted. At 50% SOC and 25 °C, the nominal starting values were $R_{0}=1.80\;m\Omega$ , $R_{1}=1.20\;m\Omega$ , $C_{1}=12.0$ kF, $R_{2}=2.60\;m\;\Omega$ , and $C_{2}=48.0$ kF. Parameter estimation minimized the measured-to-predicted voltage error Equation 10:

$$\theta^{*}=\arg\min_{\theta }\sum_{k=1}^{N}{\left[V_{\text{meas},k}-{\hat{V}}_{k}\left(\theta \right)\right]}^{2}$$ (10)

where $\theta =\left[R_{0},R_{1},C_{1},R_{2},C_{2}\right]$ . Physically constrained bounds of 0.5–8.0 m Ω for resistances and 2–100 kF for capacitances were imposed. Model accuracy was quantified by voltage root-mean-square error (RMSE) Equation 11:

$${\text{RMSE}}_{V}=\sqrt{\frac{1}{N}\sum_{k=1}^{N}{\left(V_{\text{meas},k}-{\hat{V}}_{k}\right)}^{2}}$$ (11)

A lumped thermal balance was coupled to the electrical model. Cell mass was set to $m_{c}=0.125$ kg, specific heat to $c_{p}=950$ J kg^-1^  K^−1^, exposed area to $A_{s}=0.085$ m^2^, and the convective coefficient to h=8.0 W m^-2^ K^−1^. Heat generation included irreversible polarization and reversible entropic heat Equation 12:

$$\dot{q}=I\left(U_{\text{oc}}-V_{t}\right)-I\;T\frac{\partial U_{\text{oc}}}{\partial T}$$ (12)

The cell temperature was propagated from Equation 13:

$$m_{c}c_{p}\frac{dT}{dt}=\dot{q}-hA_{s}\left(T-T_{\text{amb}}\right)$$ (13)

The operating envelope used by both application models was 2.00–3.65 V, 10%–90% SOC, and 10 °C–40 °C, matching the experimentally characterized temperature range. Charge and discharge current were limited to 1.0 C continuously and to 2.0 C for pulses of at most 10 s. A requested power command was reduced whenever the next-step prediction indicated that a voltage, SOC, current, or temperature limit would otherwise be violated.

In addition to pulse-response validation, the measured 24-h microgrid and off-grid current traces used for duty-cycle emulation were replayed through the identified model and compared with recorded terminal voltage. The 15-min energy-management command was internally propagated with a 1-s electrical/thermal integration substep so that current limiting, polarization, temperature, and SOC constraints were evaluated within each dispatch interval.

### 2.3 Microgrid and off-grid storage scenarios

Cell parameters were scaled to two pack sizes without altering the electrochemical state equations. The grid-connected microgrid pack used 240 cells in series and 140 parallel strings (240s × 140p), corresponding to 720 V nominal and 504 kWh nominal energy. The standalone off-grid pack used 192 cells in series and 42 parallel strings (192s × 42p), corresponding to 576 V nominal and 120.96 kW h nominal energy. Both systems used a bidirectional converter with 96.5% charge efficiency and 96.5% discharge efficiency at rated power; converter efficiency decreased linearly to 94.0% at 10% of rated power. The application parameters are summarized in Table 2.

**TABLE 2:** Application-level microgrid and off-grid storage configurations used for system simulation.

Parameter	Grid-connected microgrid	Standalone off-grid system
PV capacity	300 kW	90 kW
Wind capacity	150 kW	30 kW
Peak electrical load	350 kW	85 kW
Battery topology	240s × 140p	192s × 42p
Nominal battery energy	504 kW h	120.96 kW h
Converter rating	250 kW	60 kW
SOC operating window	10%–90%	10%–90%
Simulation step	15 min	15 min
Simulation horizon	365 days	365 days
Backup generation	Not used	60 kW diesel-equivalent generator
Primary control objective	Net-load smoothing and peak reduction	Renewable-first supply with reliability support

For both scenarios, 365-day renewable-generation and demand profiles were constructed at 15-min resolution from fixed diurnal and seasonal components plus zero-mean first-order autoregressive perturbations. The autoregressive coefficient was 0.85, and the stationary perturbation standard deviation was set to 8% of rated renewable power and 4% of peak load. The random seed was fixed at 42 so that all controller and chemistry comparisons used identical input time series. Annual PV and wind capacity factors were constrained to 21% and 31%, respectively, while the mean-to-peak load ratio was 0.58 for the microgrid and 0.52 for the off-grid system. These profiles were used only to generate realistic power commands for the experimentally calibrated sodium-ion battery model and were not used to infer electrochemical parameters. For the grid-connected microgrid, net load was defined by:

Robustness to the annual profile-generation assumptions was assessed by varying the autoregressive coefficient from 0.70 to 0.95, renewable perturbation standard deviation from 4% to 12% of rated renewable power, load perturbation standard deviation from 2% to 6% of peak load, and random seed across 20 realizations. A 5-min dispatch rerun was used as a temporal-resolution sensitivity check against the 15-min base case.

For the off-grid reliability check, battery energy and converter rating were independently increased by 20%, renewable capacity was increased by 20%, and the generator SOC hysteresis was changed from 20/50% to 25/55% (Equation 14). Pack-dispersion sensitivity used 500 Monte Carlo realizations with baseline cell-to-cell coefficients of variation of 1.8% for capacity and 6.2% for resistance. A literature-parameterized LFP benchmark was also run with the same power profiles and control logic to provide an application-level reference rather than a cell-level head-to-head experiment (Rostami et al., 2024).

$$P_{\text{net},k}=P_{L,k}-P_{\text{PV},k}-P_{W,k}$$ (14)

A 2-h first-order low-pass target separated the slowly varying grid exchange from the faster component assigned to the battery. The filtered reference was Equation 15:

$$P_{\text{ref},k}=P_{\text{ref},k-1}+\alpha \left(P_{\text{net},k}-P_{\text{ref},k-1}\right),\;\alpha =\frac{\Delta t}{\tau +\Delta t}$$ (15)

with τ=2h and Δt=0.25h . The unconstrained battery-power request, positive for discharge, was Equation 16:

$$P_{b,k}^{\text{req}}=P_{\text{net},k}-P_{\text{ref},k}$$ (16)

where $P_{\max}=25\text{ kW}0$ . The applied battery power was obtained using $\text{sat}\left(\cdot \right)$ with limits of ±250 kW and was reduced further whenever a predicted electrochemical or thermal constraint would be violated. This implementation produces charging during renewable surplus and discharge during rapid positive net-load excursions while avoiding an artificial price-arbitrage assumption.

For the standalone system, the instantaneous residual demand was $P_{\text{res},k}=P_{L,k}-P_{\text{PV},k}-P_{W,k}$ . Renewable generation supplied the load first. The battery then charged from renewable surplus or discharged to meet positive residual demand. The 60-kW backup generator was started when SOC reached 20% and remained on until SOC recovered to 50%; generator output was constrained to 20–60 kW to avoid inefficient low-load operation. Energy not served after application of renewable, battery, and generator limits was accumulated as unserved energy. The pack SOC update, including converter losses, was Equation 17:

$${\text{SOC}}_{k+1}={\text{SOC}}_{k}+\frac{\eta_{\text{conv}}P_{\text{ch},k}\Delta t}{E_{n}}-\frac{P_{\text{dis},k}\Delta t}{\eta_{\text{conv}}E_{n}}$$ (17)

where $E_{n}$ is the current usable nominal pack energy and $P_{\text{ch},k}$ and $P_{\text{dis},k}$ are non-negative AC-side charge and discharge powers, respectively. The two system architectures and their power-flow logic are summarized in Figure 2.

**FIGURE 2:**
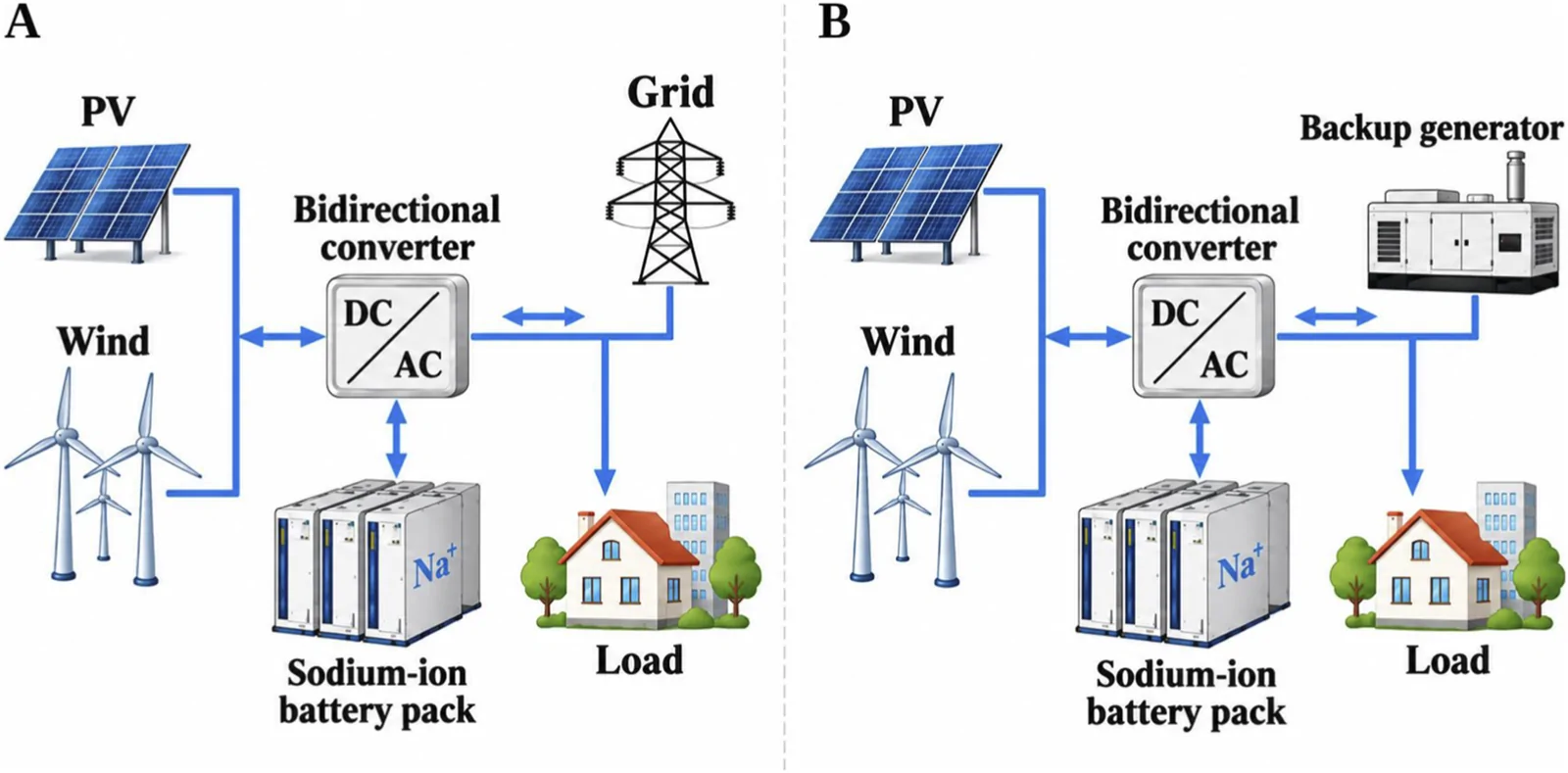
Energy-storage architectures: **(A)** Grid-connected microgrid; **(B)** Standalone off-grid system with backup generation.

### 2.4 Duty-cycle aging, performance indicators, and statistical analysis

Application-specific cell aging was performed using 24-h power traces extracted from representative high-throughput days of each annual simulation. Pack power was converted to per-cell current while preserving the pack C-rate and current sign. The microgrid trace contained frequent shallow charge-discharge reversals with a target daily throughput of 0.85 equivalent full cycles (EFCs), whereas the off-grid trace contained longer discharge intervals and renewable-recharge periods with a target daily throughput of 1.10 EFCs. The reference group was cycled at 0.50 C charge and 0.50 C discharge over the same 10%–90% SOC window. All aging tests were conducted at 25 °C until 500 EFCs or until measured capacity fell below 80% of initial capacity, whichever occurred first. An RPT was performed every 100 EFCs and comprised a 0.20 C capacity test, a 1.0 C pulse test at 50% SOC, and EIS at 50% SOC after a 60-min rest.

For duty-cycle interpretation, mean SOC, time below 20% SOC, time above 80% SOC, daily depth of discharge, RMS C-rate, peak C-rate, and charge-discharge reversal frequency were extracted from the aging traces. EIS spectra were fitted with a series ohmic resistance followed by a constant-phase-element/charge-transfer branch and a finite-length diffusion element; the same circuit and parameter bounds were used for all groups and RPTs. With 4 cells per group, a two-sided alpha of 0.05% and 80% power gave minimum detectable between-group differences of 2.8 percentage points for capacity SOH and 8.5 percentage points for resistance growth under the observed endpoint variability.

Equivalent full cycles were calculated from absolute charge throughput, with Δt expressed in hours, as Equation 18:

$$N_{\text{EFC}}=\frac{1}{2Q_{0}}\sum_{k=1}^{N}\left|I_{k}\right|\Delta t$$ (18)

with $Q_{0}$ denoting initial measured capacity. Capacity state of health and resistance growth were calculated as Equations 19, 20:

$${\text{SOH}}_{Q}=\frac{Q_{n}}{Q_{0}}\times 100$$ (19)

$$\Delta R_{0}=\frac{R_{0,n}-R_{0,0}}{R_{0,0}}\times 100$$ (20)

System-level battery round-trip efficiency was calculated over complete charge-discharge accounting intervals as Equation 21:

$$\eta_{\text{RT}}=\frac{\sum_{k}P_{\text{dis},k}\Delta t}{\sum_{k}P_{\text{ch},k}\Delta t}\times 100$$ (21)

For the off-grid case, supply adequacy was quantified using the energy-based loss-of-power-supply probability (LPSP) Equation 22:

$$\text{LPSP}=\frac{\sum_{k}E_{\text{unserved},k}}{\sum_{k}E_{\text{load},k}}\times 100$$ (22)

Renewable curtailment was normalized to the total available renewable energy Equation 23:

$$C_{\text{RE}}=\frac{\sum_{k}E_{\text{curt},k}}{\sum_{k}E_{\text{RE},k}}\times 100$$ (23)

For the microgrid, battery power-tracking performance relative to the unconstrained smoothing request was quantified by Equation 24:

$${\text{RMSE}}_{P}=\sqrt{\frac{1}{N}\sum_{k=1}^{N}{\left(P_{b,k}-P_{b,k}^{\text{req}}\right)}^{2}}$$ (24)

Initial cell-level gravimetric specific energy was calculated as Equation 25:

$$e_{\text{sp}}=\frac{E_{\text{dis}}}{m_{c}}$$ (25)

The complete endpoint set and predefined engineering interpretation thresholds are provided in Table 3. These thresholds were treated as engineering acceptance criteria and not as substitutes for statistical inference.

**TABLE 3:** Primary electrochemical and application-level performance indicators.

Indicator	Definition/criterion	Purpose
Initial specific energy	Equation 25, evaluated at 0.20 C	Baseline energy-storage capability
1 C capacity retention	$Q_{1C}/Q_{0.2C}$ ; target ≥90 %	Rate capability
Cell energy efficiency	Equation 4; target ≥90 % at 0.50 C	Electrochemical efficiency
Voltage-model error	Equation 11; target RMSE ≤30 mV	Model validity
500-EFC capacity SOH	Equation 19; target ≥80 %	Cycle durability
Resistance growth	Equation 20; lower is better	Polarization evolution
Pack round-trip efficiency	Equation 21; target ≥85 %	Application efficiency
Off-grid LPSP	Equation 22; target ≤0.1 %	Supply reliability
Renewable curtailment	Equation 23	Energy-utilization penalty
Microgrid tracking RMSE	Equation 24	Dynamic power-delivery fidelity

All reported laboratory endpoints were calculated independently for each cell before group aggregation. Aging-group results were summarized as mean ± standard deviation (n = 4 cells per group). Repeated RPT outcomes were analyzed using a linear mixed-effects model with duty-cycle group, EFC, and their interaction as fixed effects and cell identity as a random intercept. Where the interaction was significant, prespecified pairwise contrasts at 500 EFCs were adjusted by the Holm procedure. Normality of model residuals and homoscedasticity were checked before inference; when either assumption was materially violated, a rank-based repeated-measures sensitivity analysis was used. Two-sided p<0.05 was considered statistically significant, and effect sizes with 95% confidence intervals were reported alongside p values. For the annual system simulations, deterministic metrics were calculated from the common input year, and uncertainty was assessed by 1,000 bootstrap resamples of daily blocks to preserve within-day temporal dependence.

## 3 Results

### 3.1 Baseline electrochemical performance

The sodium-ion pouch cells exhibited a systematic rate-dependent decrease in delivered capacity and discharge voltage. As shown in Figure 3A, increasing the discharge rate from 0.2 to 2.0 C progressively increased voltage polarization and advanced attainment of the lower cut-off voltage. The corresponding discharge capacities decreased from approximately 4.98 A h at 0.2 C to 4.89 A h at 0.5 C, 4.72 A h at 1 C, and 4.28 A h at 2 C (Figure 3B). Capacity retention therefore declined to approximately 86% at 2 C. When the discharge rate was subsequently returned to 0.5 C, capacity recovered to approximately 4.88 A h, approaching the initial 0.5 C value and indicating that most of the high-rate capacity loss was reversible under the applied sequence (Figure 3B).

**FIGURE 3:**
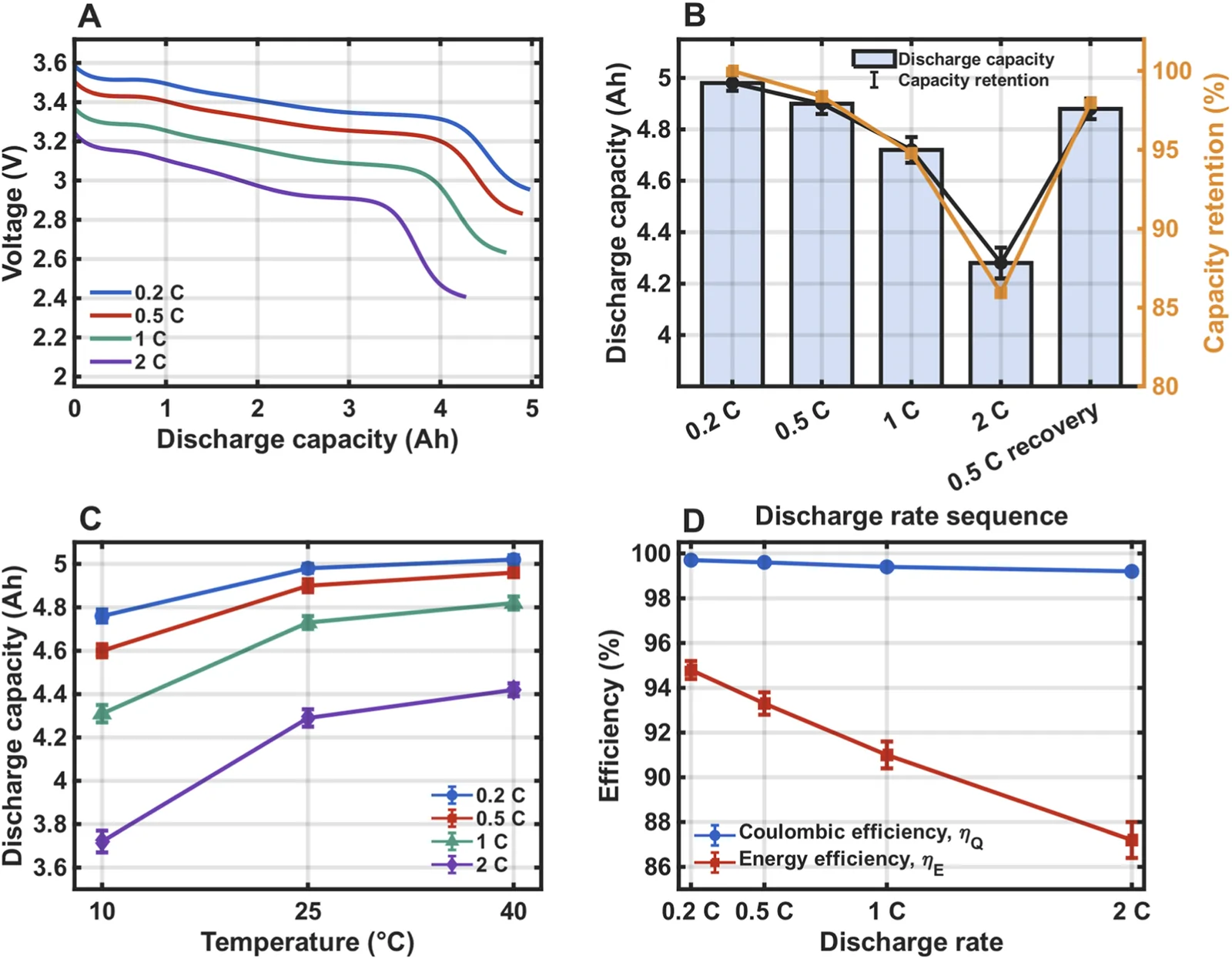
Baseline electrochemical performance of sodium-ion pouch cells. **(A)** Galvanostatic discharge profiles at 0.2–2.0 C, **(B)** rate capability and 0.5 C recovery, **(C)** temperature-dependent discharge capacity, and **(D)** Coulombic and energy efficiencies.

Temperature exerted a pronounced effect on available capacity, particularly at elevated discharge rates (Figure 3C). At 10 °C, discharge capacity decreased from approximately 4.76 A h at 0.2 C to 3.72 A h at 2 C, whereas the corresponding values at 25 °C were approximately 4.98 and 4.28 A h. Increasing the temperature to 40 °C further increased the accessible capacity, reaching approximately 5.02 A h at 0.2 C and 4.42 A h at 2 C. Coulombic efficiency remained above approximately 99% across the tested rates, whereas energy efficiency decreased from about 94.8% at 0.2 C to 87.2% at 2 C (Figure 3D). Thus, increasing discharge rate primarily reduced usable capacity and energy efficiency while Coulombic reversibility remained comparatively high.

At 2 C, model-based loss attribution of the 12.8-percentage-point deficit from unity assigned 5.9 points to instantaneous ohmic drop, 4.7 points to RC polarization, and 2.2 points to residual voltage-path and hysteretic losses. Reversible entropic heat was reported separately and was not double-counted as an electrical energy-loss term.

### 3.2 Equivalent-circuit model identification and validation

The experimentally measured open-circuit voltage increased monotonically across the validated SOC interval, from approximately 2.34 V at 10% SOC to 3.58 V at 90% SOC, and was closely reproduced by the cubic polynomial representation (Figure 4A). During pulse excitation, the second-order Thevenin model reproduced both the instantaneous voltage steps and the subsequent polarization-relaxation behavior, with the predicted terminal voltage remaining closely aligned with the measured response throughout the 60-min sequence (Figure 4B). The corresponding prediction error remained predominantly within approximately −8 to +4 mV, with transient deviations concentrated around current transitions and relaxation periods (Figure 4C).

**FIGURE 4:**
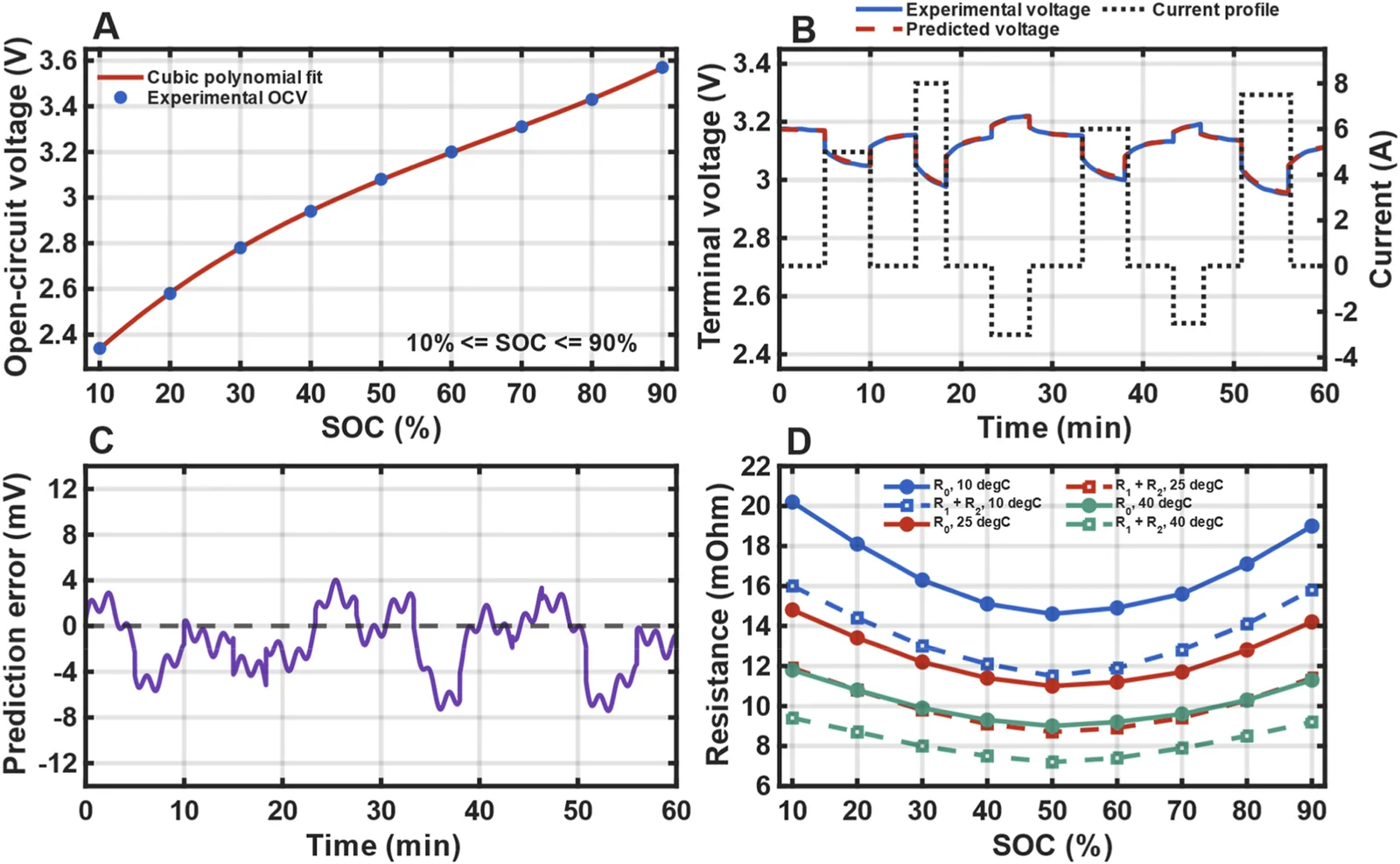
Identification and validation of the second-order Thevenin equivalent-circuit model. **(A)** Experimental and cubic-fit OCV–SOC relationship, **(B)** measured and predicted pulse-response voltage with applied current, **(C)** voltage-prediction error, and **(D)** SOC- and temperature-dependent resistance parameters.

The identified resistance parameters varied systematically with both SOC and temperature (Figure 4D). At each temperature, both the instantaneous resistance R0 and the combined polarization resistance R1 + R2 exhibited a shallow U-shaped dependence on SOC, reaching their lowest values near the mid-SOC region and increasing toward 10% and 90% SOC. Resistance decreased consistently with increasing temperature. At 50% SOC, R0 was approximately 14.5, 11.3, and 9.1 mΩ at 10, 25, and 40 °C, respectively, while R1 + R2 decreased from approximately 11.5 to 9.0 and 7.4 mΩ. These trends indicate that the identified model captured the principal SOC- and temperature-dependent variations required for the subsequent application-level simulations.

The cubic OCV fit had an overall RMSE of 2.9 mV; residuals at 10% and 90% SOC were +3.4 and −4.1 mV, respectively. Under replay of the recorded duty cycles, voltage RMSE/MAE were 7.6/5.1 mV for the microgrid trace and 9.2/6.4 mV for the off-grid trace, with maximum absolute errors of 21.8 and 25.6 mV. Across the annual simulations, minimum/maximum SOC were 10.6/89.3% for the microgrid and 10.2/89.7% for the off-grid system, while cell temperature remained between 18.7 °C and 35.1 °C; no model extrapolation beyond the validated SOC-temperature domain occurred.

### 3.3 Application-level microgrid and off-grid performance

Under grid-connected microgrid operation, renewable generation and load exhibited pronounced diurnal variability, while the battery alternated between charging and discharging to compensate short-term power imbalance (Figure 5A). The corresponding SOC trajectory remained within the prescribed 10%–90% operating window, with repeated daily excursions between the lower and upper limits (Figure 5B). Battery compensation reduced the short-term variability of the original net-load signal, producing a smoother grid-exchange profile while retaining the underlying daily demand pattern (Figure 5C). The annual microgrid simulation yielded a round-trip efficiency of 91.6%, a tracking RMSE of 18.7 kW, and renewable curtailment of 4.3% (Figure 5F).

**FIGURE 5:**
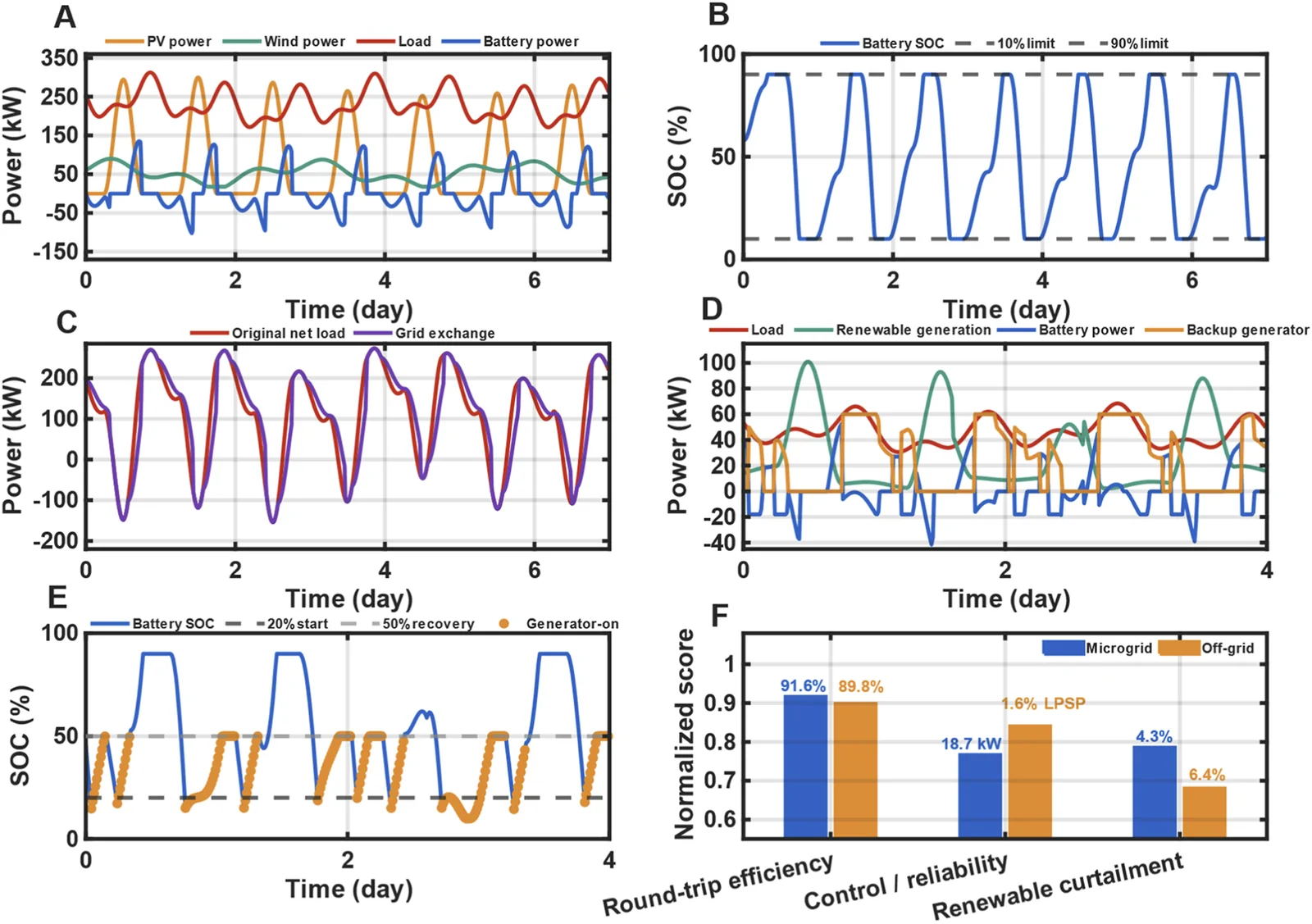
Application-level performance under microgrid and off-grid operation. **(A–C)** Microgrid power balance, SOC trajectory, and net-load smoothing; **(D,E)** off-grid power balance and SOC with backup-generation control; and **(F)** annual application-performance comparison.

In the standalone off-grid scenario, renewable generation periodically exceeded demand, whereas renewable deficits were supplied by coordinated battery discharge and backup generation (Figure 5D). Battery SOC evolved under the specified hysteresis control, with generator activation associated with the 20% SOC start threshold and continued operation until the 50% recovery threshold was reached (Figure 5E). The off-grid system achieved a round-trip efficiency of 89.8%, an LPSP of 1.6%, and renewable curtailment of 6.4% (Figure 5F). Relative to the microgrid case, the off-grid configuration therefore operated with lower round-trip efficiency and higher renewable curtailment while maintaining a low annual loss-of-power-supply probability.

Across the 20 profile-sensitivity realizations, microgrid round-trip efficiency ranged from 90.9% to 92.2%, curtailment from 3.7% to 5.1%, and tracking RMSE from 16.8 to 20.9 kW; off-grid round-trip efficiency ranged from 89.0% to 90.6%, curtailment from 5.6% to 7.5%, and LPSP from 1.1% to 2.3%. The 5-min dispatch rerun changed microgrid efficiency, curtailment, and tracking RMSE from 91.6%, 4.3%, and 18.7 kW to 91.4%, 4.4%, and 19.1 kW, while off-grid efficiency and LPSP changed from 89.8% to 1.6%–89.6% and 1.7%, respectively.

Off-grid LPSP decreased from 1.6% to 0.9% with a 20% battery-energy increase, 1.4% with a 20% converter-rating increase, 0.7% with 20% additional renewable capacity, and 1.0% with the 25/55% generator hysteresis. The pack-dispersion analysis reduced median usable pack capacity by 2.3%, increased peak temperature by 1.8 °C, reduced round-trip efficiency by 0.7 percentage points, and shortened modeled time to 80% SOH by 6.5% for the microgrid and 8.1% for the off-grid case. Under the same application profiles, the LFP benchmark produced round-trip efficiencies of 93.0% and 91.5%, 500-EFC usable-energy retention of 94.0% and 92.1%, and capacity SOH of 94.1% and 92.6% for the microgrid and off-grid duties, respectively, with an off-grid LPSP of 1.4%; these values represent a parameterized application benchmark rather than a direct experimental comparison.

### 3.4 Long-term degradation and impedance evolution

Capacity SOH declined progressively with equivalent full cycles, with degradation severity dependent on the imposed duty cycle (Figure 6A). At 500 EFC, capacity SOH decreased to approximately 85.2% for the reference group, 81.9% for the microgrid group, and 79.8% for the off-grid group; the latter reached the predefined 80% end-of-life criterion at the final test point. The corresponding resistance increase reached approximately 39%, 58%, and 74% for the reference, microgrid, and off-grid groups, respectively, at 500 EFC (Figure 6B).

**FIGURE 6:**
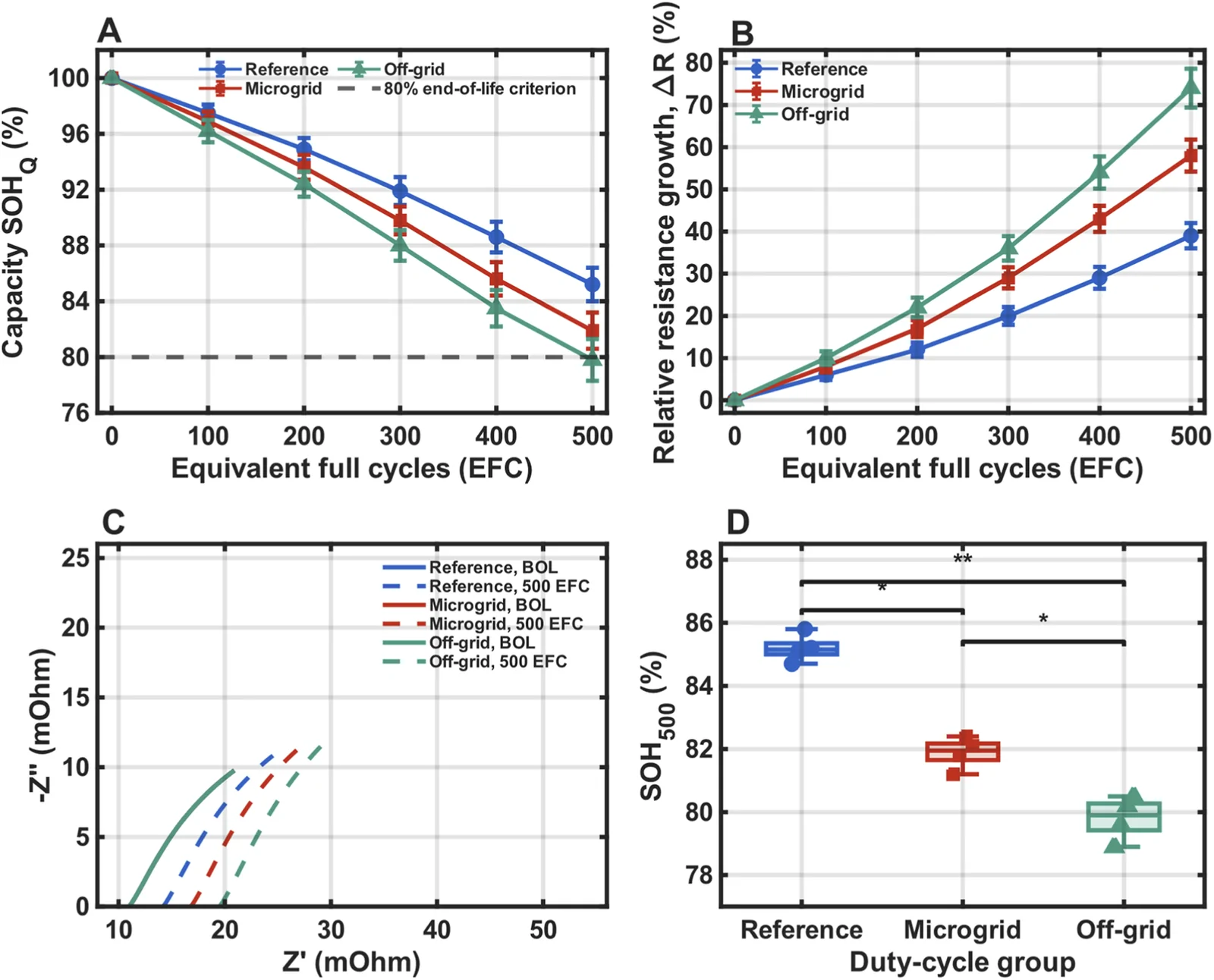
Long-term degradation behavior under reference, microgrid, and off-grid duty cycles. **(A)** Capacity state of health, **(B)** relative resistance growth, **(C)** Nyquist spectra at beginning of life and 500 EFC, and **(D)** 500-EFC capacity-SOH comparison.

Electrochemical impedance spectra exhibited a progressive rightward shift and increased polarization after long-term cycling (Figure 6C). Relative to beginning-of-life spectra, the 500-EFC response showed the smallest impedance increase for the reference condition, an intermediate increase for the microgrid duty cycle, and the largest shift for the off-grid duty cycle, consistent with the resistance-growth trajectories in Figure 6B. Endpoint capacity-SOH comparisons reproduced the same degradation ordering (Figure 6D). The significance annotations indicate pairwise differences among the duty-cycle groups at 500 EFC, with the strongest separation between the reference and off-grid conditions.

Equivalent-circuit fitting separated the impedance increase primarily into interfacial/charge-transfer and, secondarily, ohmic contributions. At 500 EFC, fitted ohmic resistance was 13.1, 13.8, and 14.6 mΩ and interfacial/charge-transfer resistance was 18.0, 24.7, and 31.9 mΩ for the reference, microgrid, and off-grid groups, respectively; the normalized diffusion-related term increased by 18%, 31%, and 46%. These full-cell fits support stronger interfacial and transport polarization under off-grid cycling but do not uniquely assign the changes to a specific electrode.

### 3.5 Standalone off-grid operational response

The representative off-grid sequence showed substantial temporal mismatch between renewable generation and electrical demand (Figure 7A), requiring frequent battery intervention to maintain the power balance. Battery power alternated between charge and discharge states in response to renewable surplus and deficit conditions, with short high-power excursions reaching approximately +50 kW during discharge and −40 kW during charging (Figure 7B). The corresponding SOC trajectory remained dynamically constrained by the backup-generation logic, with generator activation initiated near the 20% SOC threshold and continued until the battery recovered toward the 50% threshold (Figure 7C).

**FIGURE 7:**
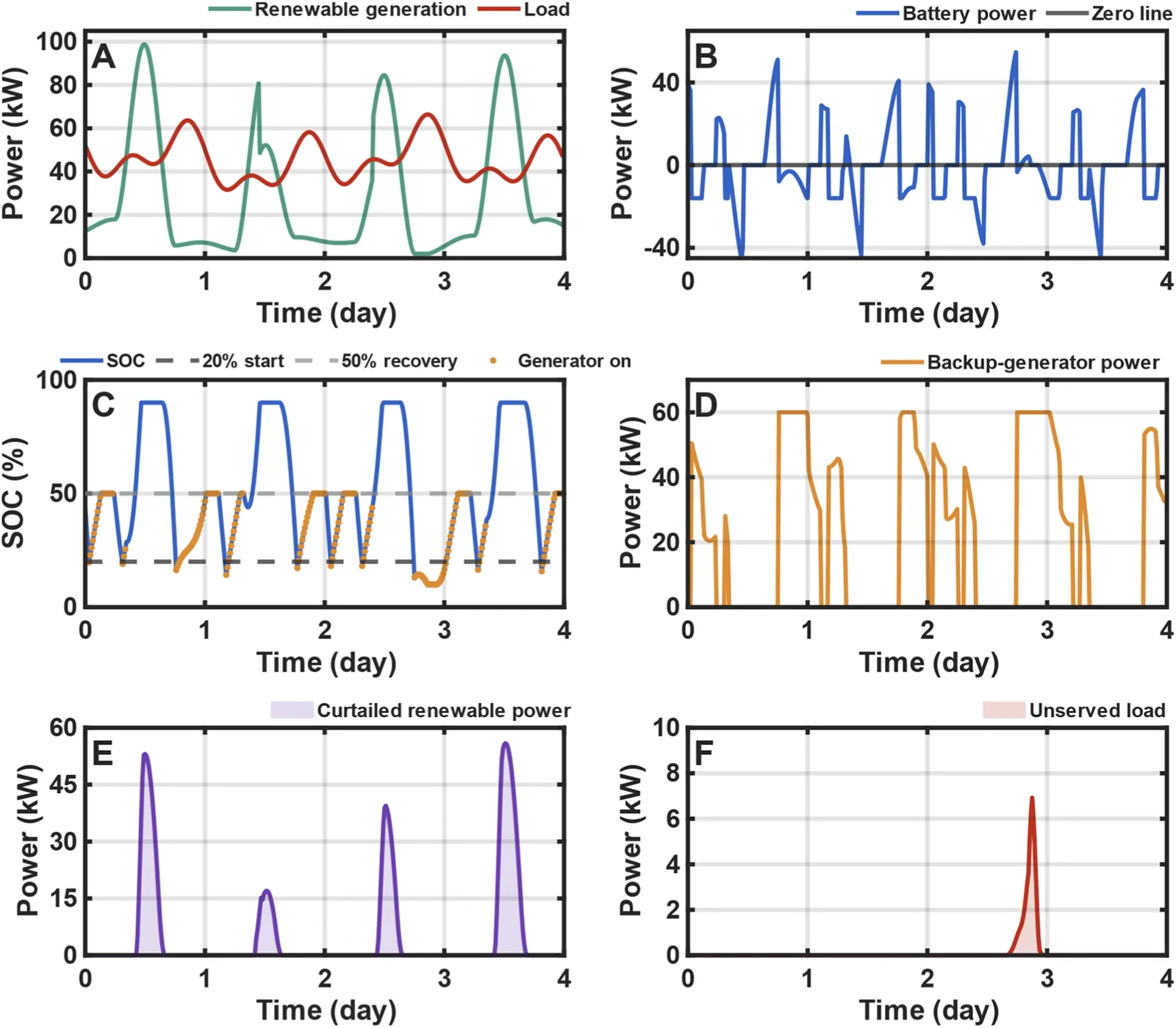
Representative standalone off-grid operation. **(A)** Renewable generation and load, **(B)** battery power, **(C)** SOC trajectory with control thresholds, **(D)** backup-generator power, **(E)** curtailed renewable power, and **(F)** unserved load.

Backup-generator output was dispatched intermittently during low-SOC periods and approached the defined upper operating range of approximately 60 kW (Figure 7D). Renewable curtailment occurred only during limited high-generation intervals, with short peaks of approximately 15–55 kW (Figure 7E). Unserved load remained negligible for most of the representative period, with only a brief isolated event reaching approximately 7 kW near the end of the third day (Figure 7F). The coordinated renewable–battery–generator strategy therefore maintained supply continuity over most of the representative horizon while limiting both curtailment and residual unmet demand.

### 3.6 Annual system-level performance

The annual operating statistics showed distinct utilization patterns between the two storage applications. The microgrid SOC distribution was concentrated predominantly between approximately 30% and 75%, whereas the off-grid system exhibited a broader distribution extending toward both the lower and upper SOC limits (Figure 8A). Daily battery energy throughput remained consistently higher in the microgrid, generally within approximately 0.38–0.50 MW h day−1, compared with about 0.10–0.16 MW h day−1 for the off-grid system (Figure 8B). Despite this higher utilization, the microgrid achieved a greater annual round-trip efficiency of 91.6%, compared with 89.8% for the off-grid configuration (Figure 8C). Renewable curtailment was also lower in the microgrid, at 4.3%, versus 6.4% under off-grid operation (Figure 8D).

**FIGURE 8:**
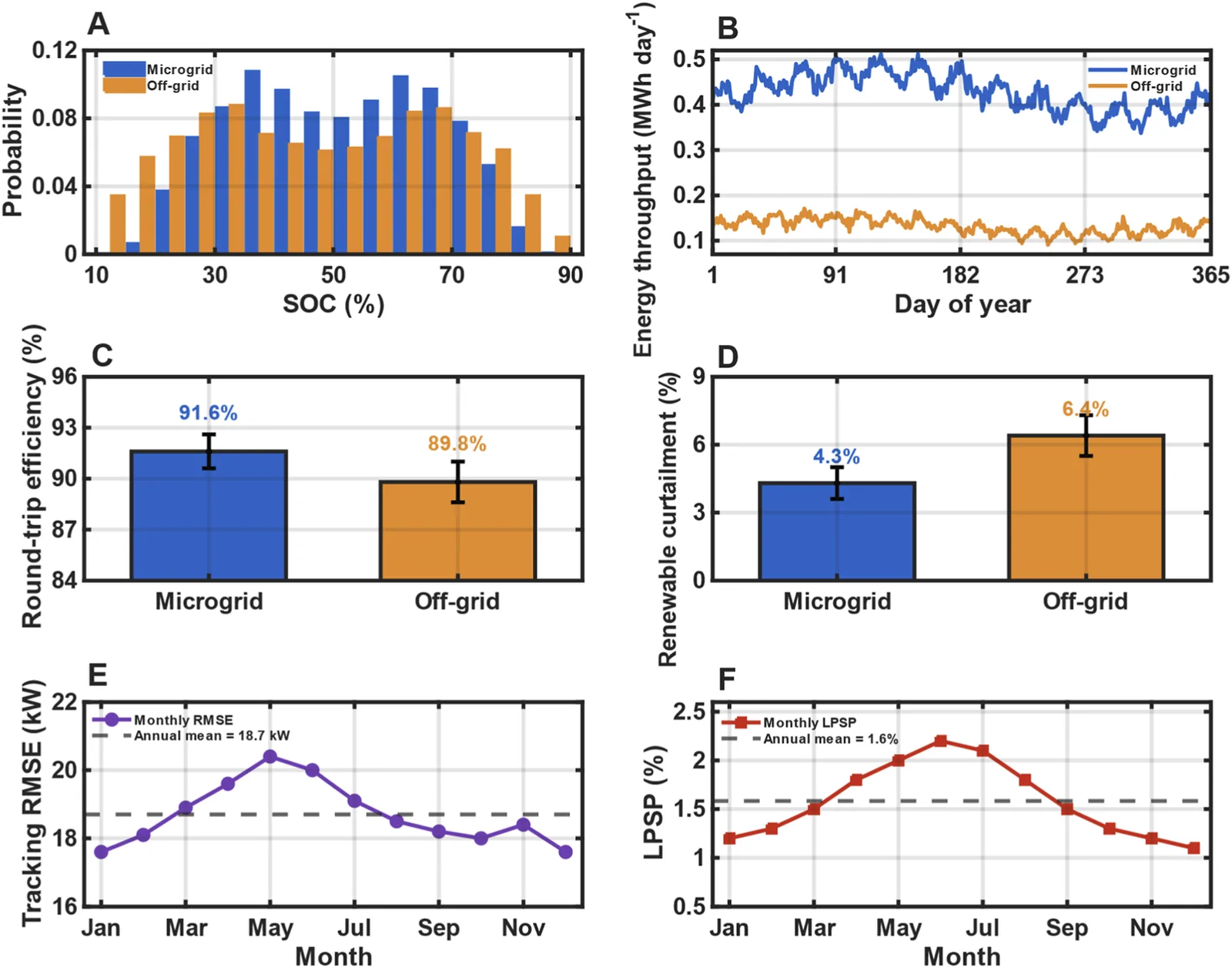
Annual system-level performance indicators. **(A)** SOC distribution, **(B)** daily battery energy throughput, **(C)** round-trip efficiency, **(D)** renewable curtailment, **(E)** monthly microgrid tracking RMSE, and **(F)** monthly off-grid LPSP.

Monthly microgrid tracking RMSE varied moderately over the year, increasing from approximately 17.5 kW in January to a maximum of about 20.4 kW in May before declining during the second half of the year; the annual mean was 18.7 kW (Figure 8E). Off-grid LPSP exhibited a stronger seasonal pattern, increasing from approximately 1.2% in January to a maximum near 2.2% in June and subsequently decreasing to approximately 1.1% by December, with an annual mean of 1.6% (Figure 8F). These annual metrics show higher conversion efficiency and lower renewable curtailment in the microgrid, while the off-grid system maintained a comparatively low supply-loss probability despite stronger seasonal variability.

### 3.7 Comparative aging and degradation assessment

The three cycling protocols produced progressively divergent degradation trajectories with increasing equivalent full cycles. Capacity SOH declined from 100% at baseline to approximately 85.2%, 81.9%, and 79.8% at 500 EFC for the reference, microgrid, and off-grid groups, respectively; only the off-grid group reached the predefined 80% end-of-life threshold at the final RPT (Figure 9A). In parallel, relative resistance growth increased to approximately 39% in the reference group, 58% under the microgrid duty cycle, and 74% under off-grid operation (Figure 9B), demonstrating progressively greater impedance-related degradation under application-specific cycling.

**FIGURE 9:**
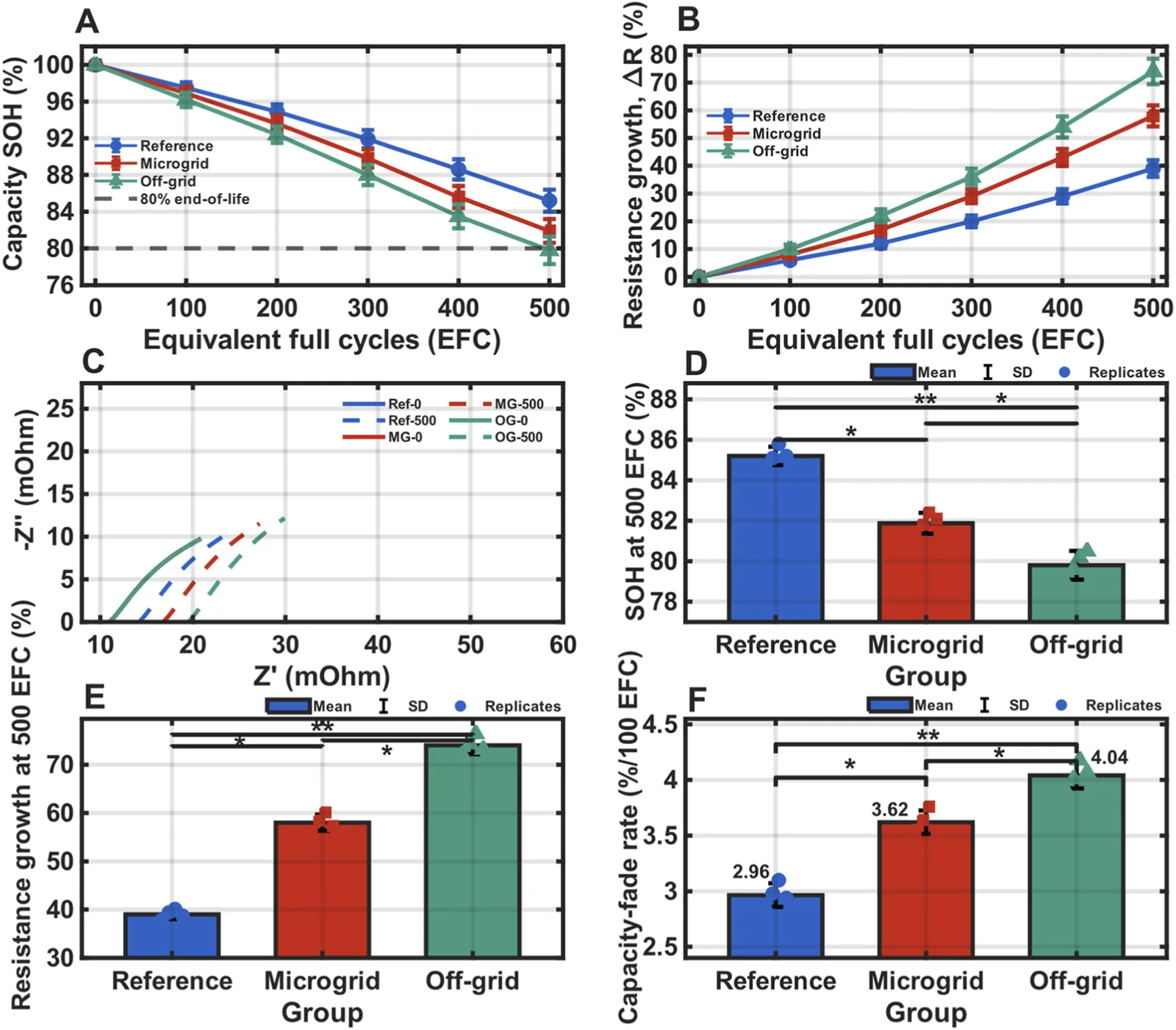
Comparative aging and degradation assessment. **(A)** Capacity SOH versus EFC, **(B)** resistance growth versus EFC, **(C)** Nyquist spectra at 0 and 500 EFC, **(D)** 500-EFC capacity comparison, **(E)** 500-EFC resistance comparison, and **(F)** capacity-fade rate across reference, microgrid, and off-grid groups.

The electrochemical impedance spectra showed corresponding duty-cycle-dependent shifts after 500 EFC (Figure 9C). Relative to the beginning-of-life spectra, the 500-EFC curves shifted toward higher real impedance, with the smallest displacement in the reference group and the largest in the off-grid group. Endpoint analysis confirmed separation among the three duty-cycle groups for capacity SOH (Figure 9D) and resistance growth (Figure 9E), as indicated by the pairwise significance brackets. The calculated capacity-fade rate increased from 2.96% per 100 EFC for reference cycling to 3.62% per 100 EFC for the microgrid duty cycle and 4.04% per 100 EFC for the off-grid duty cycle (Figure 9F). Collectively, the capacity, resistance, impedance, and fade-rate endpoints showed progressively greater degradation from reference cycling to microgrid and off-grid duty cycles.

Trace descriptors confirmed that the application groups differed in more than nominal pack size: mean SOC was 52.4% for microgrid and 46.7% for off-grid cycling; time below 20% SOC was 4.8% and 9.6%, time above 80% SOC was 6.3% and 10.8%, daily depth of discharge was 41.6% and 61.3%, RMS C-rate was 0.32 and 0.41, and peak C-rate was 0.92 and 0.97, respectively. The faster off-grid degradation is therefore interpreted as the combined effect of deeper excursions, longer low-SOC residence, higher current severity, and greater throughput rather than duty-cycle shape alone.

At fixed microgrid duty cycle, the N/P balancing check predicted 500-EFC capacity SOH of 80.7%, 81.9%, and 82.5% and resistance growth of 65%, 58%, and 54% for N/P ratios of 1.05, 1.10, and 1.15, respectively, indicating moderate but non-negligible sensitivity to electrode balance. The observed reference-to-off-grid endpoint differences of 5.4 percentage points in capacity SOH and 35 percentage points in resistance growth exceeded the calculated minimum detectable effects, although the small group size still limits precision.

Mechanistically, repeated wide SOC swings, prolonged low-SOC residence, and high-current events can increase hard-carbon interphase growth and charge-transfer polarization, while repeated layered-oxide cycling can promote structural rearrangement and transition-metal-related cathode degradation (J et al., 2022; An et al., 2024; Zheng et al., 2024; Zeng et al., 2025; Li et al., 2025; Xie et al., 2026). Because the present measurements are full-cell EIS without electrode-specific post-mortem analysis, the relative cathode and anode contributions cannot be uniquely separated. Aging was performed at 25 °C; higher temperature would be expected to accelerate interfacial side reactions, whereas lower temperature would increase polarization, so the exact seasonal ranking requires direct temperature-dependent aging tests. The 79.8% SOH value at 500 EFC should therefore be interpreted as a group mean for the tested cells, temperature, SOC window, and duty protocol, not as a universal cycle-life limit for sodium-ion batteries.

## 4 Conclusion

The present study integrated cell-level electrochemical characterization, a second-order Thevenin electrochemical-thermal model, and application-specific simulations to evaluate sodium-ion pouch cells for grid-connected microgrid and standalone off-grid storage. Baseline testing showed that increasing discharge rate increased polarization and reduced usable capacity and energy efficiency, whereas coulombic efficiency remained above approximately 99% across the tested rates. The experimentally fitted model reproduced the OCV-SOC relationship and pulse-response behavior with millivolt-scale prediction errors while capturing the expected dependence of ohmic and polarization resistance on SOC and temperature. At the system level, the microgrid configuration achieved a round-trip efficiency of 91.6%, a tracking RMSE of 18.7 kW, and renewable curtailment of 4.3%, whereas the off-grid configuration achieved a round-trip efficiency of 89.8%, an annual LPSP of 1.6%, and renewable curtailment of 6.4%.

Long-term cycling demonstrated a clear dependence of degradation on application duty cycle. At 500 EFC, capacity SOH was approximately 85.2% for reference cycling, 81.9% for the microgrid duty cycle, and 79.8% for the off-grid duty cycle, accompanied by relative resistance increases of approximately 39%, 58%, and 74%, respectively. The corresponding capacity-fade rates were 2.96%, 3.62%, and 4.04% per 100 EFC. These results indicate that the same sodium-ion chemistry can provide effective short-duration balancing and reliability support in both stationary applications, but that the deeper and more sustained power excursions associated with standalone operation impose a greater aging penalty than grid-connected microgrid service under the conditions investigated.

The study was conducted with a limited laboratory population of 24 pouch cells, including four aging replicates per duty-cycle group, and the aging horizon was restricted to 500 EFC or the 80% SOH criterion. The annual renewable-generation and load profiles were generated from prescribed diurnal, seasonal, and autoregressive components rather than measured field data, and pack-level simulations assumed scaled cell behavior without explicitly resolving cell-to-cell heterogeneity, module-level thermal gradients, balancing losses, or long-term calendar aging. The converter and backup-generator representations were also simplified relative to full hardware implementations. Consequently, the reported application metrics should be interpreted as controlled comparative results for the defined parameter set rather than direct field-performance estimates. Calendar aging was not included; because the microgrid and off-grid groups accumulated EFC at different daily rates and occupied different SOC ranges, calendar time, dwell SOC, and temperature could alter long-term separation between the groups. Likewise, the 25 °C aging condition, four-cell group size, and 500-EFC horizon constrain extrapolation beyond the tested protocol. The LFP benchmark provides context only and does not replace a matched experimental comparison.

Future investigations should extend cycling beyond 500 EFC and incorporate calendar-aging periods, larger cell populations, and module- or pack-scale validation under measured microgrid and off-grid power profiles. Electrochemical-thermal modeling should be expanded to include cell-to-cell variability, spatial temperature gradients, balancing behavior, and degradation-state-dependent parameter updating. Multi-year stochastic simulations and hardware-in-the-loop or field demonstrations would enable evaluation of reliability under a broader range of renewable and demand conditions. Comparative studies with alternative sodium-ion chemistries and established lithium-ion systems, together with economic and life-cycle assessment, would further clarify the operating domains in which sodium-ion storage provides the most favorable technical and sustainability trade-offs.

## Data Availability

The raw data supporting the conclusions of this article will be made available by the authors, without undue reservation.

## References

[B1] Alvira D. Antorán D. Manyà J. J. (2022). Plant-derived hard carbon as anode for sodium-ion batteries: a comprehensive review to guide interdisciplinary research. Chem. Eng. J. 447, 137468. 10.1016/j.cej.2022.137468

[B2] An S. Karger L. Dreyer S. L. Hu Y. Barbosa E. Zhang R. (2024). Improving cycling performance of the NaNiO2 cathode in sodium-ion batteries by titanium substitution. Mater. Futur. 3 (3), 035103. 10.1088/2752-5724/ad5faa

[B3] Batuecas E. Martínez-Cisneros C. S. Serrano D. Várez A. (2024). Life cycle assessment of lab-scale solid sodium-ion batteries: a sustainable alternative to liquid lithium-ion batteries. J. Energy Storage 80, 110355. 10.1016/j.est.2023.110355

[B4] Boujdaini L. E. Mezrhab A. Moussaoui M. A. Jurado F. Vera D. (2022). Sizing of a stand-alone PV-wind-battery-diesel hybrid energy system and optimal combination using a particle swarm optimization algorithm. Electr. Eng. 104, 3339–3359. 10.1007/s00202-022-01529-0

[B5] Chaudhary G. Lamb J. J. Burheim O. S. Austbø B. (2021). Review of energy storage and energy management system control strategies in microgrids. Energies 14 (16), 4929. 10.3390/en14164929

[B6] Chen X. Zheng Y. Zheng Y. Zhang C. Li S. Li J. (2019). High-performance sodium-ion batteries with a hard carbon anode: transition from the half-cell to full-cell perspective. Nanoscale 11, 22196–22205. 10.1039/c9nr07545c 31742294

[B7] Deguenon L. Yamegueu D. kadri S. M. Gomna A. (2023). Overcoming the challenges of integrating variable renewable energy to the grid: a comprehensive review of electrochemical battery storage systems. J. Power Sources 580, 233343. 10.1016/j.jpowsour.2023.233343

[B8] Gao Y. Zhang H. Peng J. Li L. Xiao Y. Li L. (2024). A 30-year overview of sodium-ion batteries. Carbon Energy 6 (6), e464. 10.1002/cey2.464

[B9] Javed M. S. Jurasz J. McPherson M. Dai Y. Ma T. (2022). Quantitative evaluation of renewable-energy-based remote microgrids: curtailment, load shifting, and reliability. Renew. Sustain. Energy Rev. 164, 112516. 10.1016/j.rser.2022.112516

[B10] Jin Y. Le P, M. L. Gao P. Xu Y. Xiao B. Engelhard M. H. (2022). Low-solvation electrolytes for high-voltage sodium-ion batteries. Nat. Energy 7, 718–725. 10.1038/s41560-022-01055-0

[B11] Lai X. Chen J. Chen Q. Han X. Lu L. Dai H. (2023). Comprehensive assessment of carbon emissions and environmental impacts of sodium-ion batteries and lithium-ion batteries at the manufacturing stage. J. Clean. Prod. 423, 138674. 10.1016/j.jclepro.2023.138674

[B12] Lamb J. Manthiram A. (2020). Synthesis control of layered oxide cathodes for sodium-ion batteries: a necessary step toward practicality. Chem. Mater. 32 (19), 8431–8441. 10.1021/acs.chemmater.0c02435

[B13] Li Y. Vasileiadis A. Zhou Q. Lu Y. Meng Q. Li Y. (2024). Origin of fast charging in hard carbon anodes. Nat. Energy 9, 134–142. 10.1038/s41560-023-01414-5

[B14] Li W. Zhang H. Xie L. Fan Z. Yang T. Hua W. (2025). Advancements in the realm of structural engineering for sodium-ion batteries via elemental doping: a focus on P2-phase nickel-manganese layered oxides. Battery Energy 4 (3), e20240052. 10.1002/bte2.20240052

[B15] Lipu M.S. H. Ansari S. Miah S. Hasan K. Meraj S. T. Faisal M. (2022). A review of controllers and optimizations based scheduling operation for battery energy storage system towards decarbonization in microgrid: challenges and future directions. J. Clean. Prod. 360, 132188. 10.1016/j.jclepro.2022.132188

[B16] Liu Q. Xu R. Mu D. Tan G. Gao H. Li N. (2022). Progress in electrolyte and interface of hard carbon and graphite anode for sodium-ion battery. Carbon Energy 4(3). 458–479. 10.1002/cey2.120

[B17] Meng J. Jia G. Yang H. Wang M. (2022). Recent advances for SEI of hard carbon anode in sodium-ion batteries: a mini review. Front. Chem. 10, 986541. 10.3389/fchem.2022.986541 36204151 PMC9531120

[B18] Narayan Singh A. Islam M. Meena A. Faizan M. Faizan M. (2023). Unleashing the potential of sodium-ion batteries: current state and future directions for sustainable energy storage. Adv. Funct. Mater. 33 (46), 2304617. 10.1002/adfm.202304617

[B19] Peters J. F. Baumann M. Binder J. R. Weil M. (2021). On the environmental competitiveness of sodium-ion batteries under a full life cycle perspective - a cell-chemistry specific modelling approach. Sustain. Energy and Fuels 5, 6414–6429. 10.1039/d1se01292d

[B20] Phogat P. Dey S. Wan M. , Comprehensive review of sodium-ion batteries: principles, materials, performance, challenges, and future perspectives. Mater. Sci. Eng. B, (2025). 312: p. 117870, 10.1016/j.mseb.2024.117870

[B21] Rostami H. Valio J. Tynjälä P. Lassi U. Suominen P. (2024). Life cycle of LiFePO4 batteries: production, recycling, and market trends. ChemPhysChem 25 (24), e202400459. 10.1002/cphc.202400459 39264359 PMC11648842

[B22] Shao W. Shi H. Jian X. Wu Z.Sh. Hu F. (2022). Hard-carbon anodes for sodium-ion batteries: recent status and challenging perspectives. Adv. Energy Sustain. Res. 3 (7), 2200009. 10.1002/aesr.202200009

[B23] Wan Y. Liu Y. Chao D. Li W. Zhao D. (2023). Recent advances in hard carbon anodes with high initial coulombic efficiency for sodium-ion batteries. Nano Mater. Sci. 5 (2), 189–201. 10.1016/j.nanoms.2022.02.001

[B24] Wang Z. Chen S. Qiu J. Li J. Zhao W. Ji Y. (2023). Full-cell presodiation strategy to enable high-performance Na-ion batteries. Adv. Energy Mater. 13 (45), 2302514. 10.1002/aenm.202302514

[B25] Wang X. Zhang Q. Zhao C. Li H. Zhang B. Zeng G. (2024). Achieving a high-performance sodium-ion pouch cell by regulating intergrowth structures in a layered oxide cathode with anionic redox. Nat. Energy 9, 184–196. 10.1038/s41560-023-01425-2

[B26] Wickerts S. Arvidsson R. Nordelöf A. Svanström M. Johansson P. (2024). Prospective life cycle assessment of sodium-ion batteries made from abundant elements. J. Industrial Ecol. 28 (1), 116–129. 10.1111/jiec.13452

[B27] Wu C. Yang Y. Zhang Y. Xu H. He X. Wu X. (2024). Hard carbon for sodium-ion batteries: progress, strategies and future perspective. Chem. Sci. 15, 6244–6268. 10.1039/d4sc00734d 38699270 PMC11062112

[B28] Wu Y. Shuang W. Wang Y. Chen F. Tang S. Wu X. L. (2024). Recent progress in sodium-ion batteries: advanced materials, reaction mechanisms and energy applications. Electrochem. Energy Rev. 7, 17. 10.1007/s41918-024-00215-y

[B29] Xie Y. Wang Y. Yamauchi Y. Kim M. Yuan F. He Y. (2026). Synergistic ultramicropore-confined and electronic-state modulation strategies in sustainable lignin-derived hard carbon for robust sodium-ion batteries. Research 9, 1039. 10.34133/research.1039 41551911 PMC12804597

[B30] Yao A. Benson S, M. Chueh W, C. , Critically assessing sodium-ion technology roadmaps and scenarios for techno-economic competitiveness against lithium-ion batteries. Nat. Energy, (2025). 10. 404–416. 10.1038/s41560-024-01701-9

[B31] Zeng C. Zhang Z. Peng J. Qiao J. Pan Q. Zheng F. (2025). The synthesis effects on the performance of P2-Na0.6Li0.27Mn0.73O2 cathode material for sodium-ion batteries. Battery Energy 4 (2), e70000. 10.1002/bte2.70000

[B32] Zhao L. F. Hu Z. Lai W. H. Tao Y. Peng J. Miao Z. C. (2021). Hard carbon anodes: fundamental understanding and commercial perspectives for Na-ion batteries beyond Li-ion and K-ion counterparts. Adv. Energy Mater. 11 (1), 2002704. 10.1002/aenm.202002704

[B33] Zheng H. Zeng J. Wan X. Song X. Peng C. Wang J. (2024). ICE optimization strategies of hard carbon anode for sodium-ion batteries: from the perspective of material synthesis. Mater. Futur. 3 (3), 032102. 10.1088/2752-5724/ad5d7f

[B34] Zuo W. Innocenti A. Zarrabeitia M. Bresser D. Yang Y. Passerini S. (2023). Layered oxide cathodes for sodium-ion batteries: storage mechanism, electrochemistry, and techno-economics. Accounts Chem. Res. 56 (3), 284–296. 10.1021/acs.accounts.2c00690 36696961 PMC9910041

